# Novel Heredity Basis of the Four-Horn Phenotype in Sheep Using Genome-Wide Sequence Data

**DOI:** 10.3390/ani13203166

**Published:** 2023-10-10

**Authors:** Haoyuan Zhang, Pu Yang, Chengli Liu, Yuehui Ma, Yanguo Han, Yan Zeng, Yongfu Huang, Yongju Zhao, Zhongquan Zhao, Xiaohong He, Guangxin E

**Affiliations:** 1 College of Animal Science and Technology, Southwest University, Chongqing 400715, China; 2 Institute of Animal Husbandry and Veterinary Medicine, Chinese Academy of Agricultural Sciences, Beijing 100097, China

**Keywords:** four-horn phenotype, chondrocyte, genome-wide association study

## Abstract

**Simple Summary:**

We performed a genome-wide association study of 100 sheep genomes to investigate the hereditary basis of the four-horn phenotype (FHP) in sheep. The results revealed that a total of 3 InDels (e.g., CHR2: g.133,742,709delA and g.133,743,215insC) and 14 SNPs (e.g., CHR2: g.133,727,513C > T and CHR16: g.40,351,378G > A) in CHR2 and CHR16 were significantly associated with the four-horn trait. Furthermore, *HOXD1* and *ADAMTS12* annotated from both CHR sequences were the key candidate gene loci contributing to FHP in sheep. This study provided a novel understanding of the Mendelian genetic basis for four horns in sheep.

**Abstract:**

Horns are an important breeding trait for sheep. However, no widely recognized viewpoint on the regulatory genes and mechanisms of horns is available, and the genetic basis of the four-horn phenotype (FHP) is unclear. This work conducted a genome-wide association study with 100 sheep genomes from multiple breeds to investigate the genetic basis of the FHP. The results revealed three significant associations (corrected as *p* < 1.64 × 10^−8^) of the InDels (CHR2: g.133,742,709delA, g.133,743,215insC, and g.133,743,940delT) for FHP in the intergenic sequence (IGS) between the *MTX2* and the *LOC105609047* of CHR2. Moreover, 14 significant associations (corrected as *p* < 1.42 × 10^−9^) of SNPs with the FHP phenotype were identified in CHR2 and CHR16, including five (e.g., CHR16: g.40,351,378G > A and g.40,352,577G > A) located in the intron of the *ADAMTS12* gene, eight (e.g., CHR2: g.133,727,513C > T and g.133,732,145T > G) in the IGS between *MTX2* and *LOC105609047*, and only one (CHR2: g.133,930,761A > G) in the IGS between *HOXD1* and *MTX2*. Obvious divergence was also observed in genotype patterns between the FHP and others (two horns and hornless) in the *HOXD1* and *ADAMTS12* gene regions. An extremely significant linkage also occurred between Loci I and Loci II within 100 individuals (LD = −156.02186, *p* < 0.00001). In summary, our study indicated that the genomic sequences from CHR2 and CHR16 contributed to the FHP in sheep, specifically the key candidate genes *HOXD1* and *ADAMTS12*. These results improved our understanding of the Mendelian genetic basis of the FHP in sheep.

## 1. Introduction

Sheep are important domestic animals that play an important role in regional economic development and meat supply security [1]. To date, most studies on sheep have focused on the phylogenetic history of the population [2], the genetic diversity of indigenous sheep [3], and the genetic basis of important economic traits [4,5].

Horns are extensively considered to be an important Mendelian trait in many ruminants, and hornless herbivores accord better with the requirements of current intensive animal husbandry management [6]. Numerous studies have increased our understanding of the formation basis of horn traits and the genetic basis of hornless phenotypes in ruminants [7,8]. For example, *RXFP2* expression is reportedly upregulated significantly in horn buds, indicating that *RXFP2* may be a key gene in the regulation of horn development [9]. Moreover, a 1.8 kb insertion in the 3′-UTR of *RXFP2* contributes to the hornless phenotype in multiple domestic sheep breeds [10].

The four-horn phenotype (FHP) of sheep is a major research topic. The genetic locus of the FHP is located on chromosome (CHR) 2 [11,12]. The genomic markers associated with this phenotype have also been narrowed down to the HOXD gene family member genome regions of CHR2 in Sishui fur sheep [13]. However, previous works lack precise localization and overlook genetic background interference because of limited genetic markers (Illumina OvineSNP50 BeadChip) or a single population with four horns [11,13].

In the present study, an association analysis of multiple breeds and large samples was performed to identify FHP-associated genes with high-depth whole-genome sequencing. Our results can help further elucidate the Mendelian genetic basis of the FHP in sheep.

## 2. Materials and Methods

A total of 88 publicly available sheep genome datasets were downloaded from the NCBI SRA Database (PRJNA624020, Table S1), including 10 four-horn sheep (Sishui fur sheep), 26 two-horn sheep (e.g., small-tailed Han sheep), and 52 polled sheep (e.g., Hu sheep and large-tailed Han sheep). The blood samples of 12 Mongolian sheep with four horns were supported by the Institute of Animal Sciences of CAAS. Genomic DNA was extracted following a standard phenol–chloroform protocol (Sambrook and Russel, 2001). Sequencing libraries were constructed with Annoroad^®^ Universal DNA Library Prep Kit v2.0 (Illumina^®^, 15,026,486 Rev. C, San Diego, CA, USA). Sequencing was performed using the BGI DNBSEQ-T7 platform (Beijing Genomics Institution, Shenzhen, China) with > 10× per individual.

Fastp (v0.20.0) [14] was used with the parameter ”-w 15 --cut_window_size 4 --cut_mean_quality 15 -5 3 -3 3 --length_required 40” to obtain high-quality (HQ) reads for raw data. HQ reads were aligned to the reference genome (GCF_016772045.1) using BWA (v0.7.17) [15] with the MEM algorithm. Finally, the picard software (v2.27.0) (https://github.com/broadinStitute/picard (accessed on 1 June 2022)) was used to remove PCR duplicates. Single-nucleotide polymorphisms (SNPs) and insertion deletions (InDels) were identified and filtered using GATK (v4.2.4.1) [16] with custom parameters (“QD < 2.0, MQ < 40.0, FS > 60.0, SOR > 3.0, MQRank-Sum < −12.5, ReadPosRankSum < −8.0” for SNPs; “QD < 2.0, FS > 200.0, SOR > 10.0, MQRankSum < −12.5, ReadPosRankSum < −8.0” for InDels). The data quality control of the SNP and InDel datasets was processed using PLINK v1.90 (https://www.cog-genomics.org/plink/1.9/ (accessed on 1 June 2023)) with a minor allele frequency (MAF) < 0.05 and a call rate < 90% removed, and principal component analysis was performed with the obtained SNPs using PLINK v1.90.

A genome-wide association study (GWAS) was performed using PLINK with a general linear model. All animals were divided into three categories according to horn number: four horns (4), two horns (2), and polled (0). *p*-values were corrected using the Bonferroni multiple test, and the significance threshold was defined as 0.05/*N*, where *N* represents the number of genome variants for SNPs or InDels. The GeneCards database (https://www.genecards.org/ (accessed on 28 September 2023)) was used to display Gene Ontology (GO) analyses for candidate genes. Genotype patterns of genes were determined with RectChr v1.36 (https://github.com/BGI-shenzhen/RectChr (accessed on 1 August 2023)). The haplotypes of selected SNPs were constructed with DnaSP 6.12.03 (http://www.ub.edu/dnasp/ (accessed on 1 June 2023)). Linkage disequilibrium and pairwise differences (*F_ST_*) of haplotypes were analyzed with Arlequin v3.5.2.2 (http://cmpg.unibe.ch/software/arlequin35/ (accessed on 1 June 2023)).

## 3. Results and Discussion

**Figure 1:**
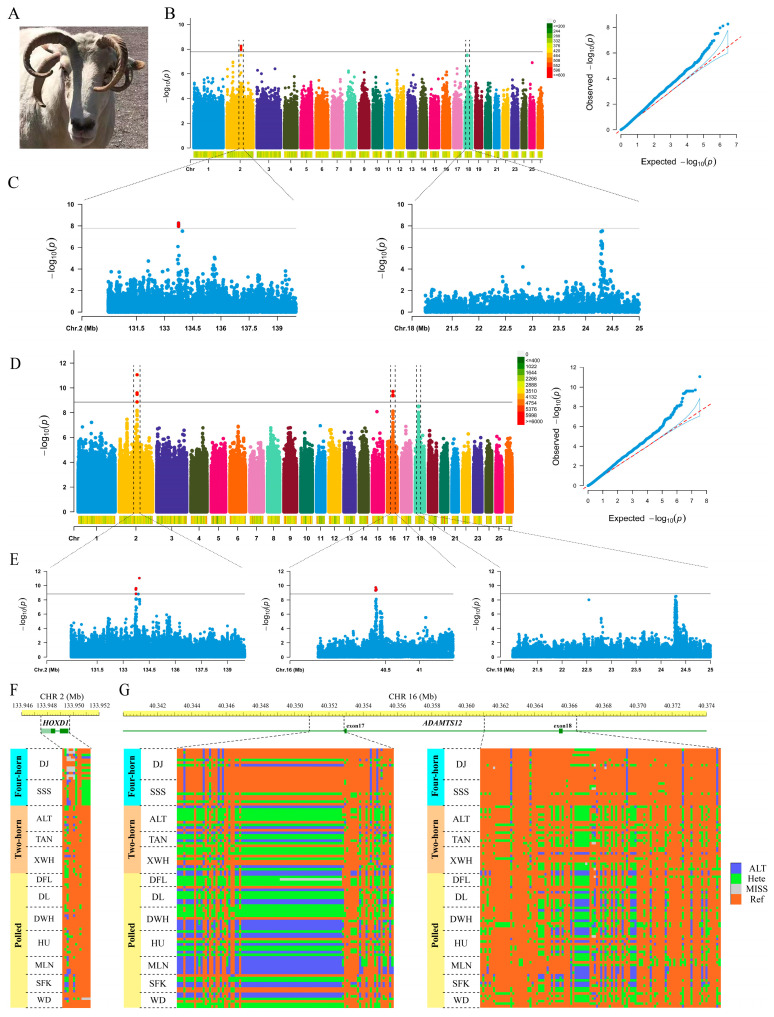
Genome-wide association study (GWAS) of the four-horn trait with multiple breeds based on SNP and InDel datasets. (**A**) FHP of Mongolian sheep. (**B**) Manhattan plot and quantile–quantile plot of results for GWAS based on InDels. (**C**) Manhattan plots of 2 regions in CHR2 (130.0–140.0 Mb) and CHR18 (21.0–25.0 Mb) based on InDels. (**D**) Manhattan plot and quantile–quantile plot of results for GWAS based on SNPs. (**E**) Manhattan plots of 3 regions in CHR2 (130.0–140.0 Mb), CHR16 (39.5–41.5 Mb), and CHR18 (21.0–25.0 Mb) based on SNPs. (**F**) Genotype patterns of *HOXD1* among the 3 categories. (**G**) Genotype patterns of *ADAMTS12* among the 3 categories.

An approximately 310.79 Gb dataset was generated from 12 sequenced individuals and uploaded to the Sequence Read Archives (PRJNA1003686). The sequencing depth of the 12 samples ranged from 10.43- to 18.04-fold (Table S2). First, a total of 3,042,046 InDels were obtained after filtering from all 100 individuals, and the GWAS results revealed that three InDels (CHR2: g.133,742,709delA, g.133,743,215insC, and g.133,743,940delT) exceeded the Bonferroni significance threshold (corrected as *p* < 1.64 × 10^−8^; Figure 1B). Based on the physical location of the chromosomes, the three InDels were distributed in the intergenic sequence (IGS) between *MTX2* and *LOC105609047*. In fact, *MTX2* was confirmed to be related to bone development. For example, the splice site variant of the *MTX2* gene (c.543 + 1G > T) can cause human mandibuloacral dysplasia, which is characterized by postnatal growth retardation, hypotonia, and dysmorphic facial features [17,18].

Furthermore, a total of 35,120,641 SNPs were obtained after filtering from all animals for further GWAS analysis. The GWAS results revealed that 14 SNPs exceeded the Bonferroni significance threshold (corrected as *p* < 1.42 × 10^−9^; Figure 1D). Among them, five SNPs (CHR16: g.40,351,378G > A, g.40,352,577G > A, g.40,354,371C > T, g.40,354,900G > A, and g.40,363,930T > G) were located in the intron of the *ADAMTS12* gene, one SNP (CHR2: g.133,930,761A > G) was located in the IGS between *HOXD1* and *MTX2*, and eight SNPs (CHR2: g.133,727,513C > T, g.133,732,145T > G, g.133,732,461G > A, g.133,734,690C > T, g.133,736,440C > T, g.133,737,513T > A, g.133,738,352A > G, and g.133,741,832G > T) were distributed in the IGS between *MTX2* and *LOC105609047*.

Obviously, the SNP (CHR2: g.133,930,761A > G) with the highest associated signal value was located in the region between *HOXD1* and *MTX2* on CHR2, consistent with previous findings [11,12]. Near the threshold line, a series of high-signal SNPs was annotated to the introns and 3′-UTR of *HOXD1*. The genotype patterns of *HOXD1* showed obvious differences between four-horn breeds and others (Figure 1F). *HOXD1* haploinsufficiency leads to the division of horn sprout progeny [19]. In particular, a number of studies have shown that the HOXD gene family of CHR2 may be related to the FHP [11,12,20]. In fact, HOXD family genes have been identified as playing an important role in vertebrate limb development. For example, the targeted disruption of exon 2 in *HOXD10* leads to changes in the hind limb, such as an anterior shift in the position of the patella. These changes contribute to defects in mouse locomotion [21]. *HOXD11* is highly expressed in the carpal, tarsal, and digital regions during limb development in mammals [22]. *HOXD11* has also been demonstrated to regulate chondrocyte differentiation at an early step in the zeugopods of mice [23]. An SNP in *HOXD12* (c.1359G > T) induces a change from alanine to serine and thus causes an abnormal limb phenotype in mice with macrodactyly [24]. Severe brachydactyly with metacarpal-to-carpal transformation is also underlined by a missense mutation (c.938C > G) in *HOXD13* [25].

Additionally, several SNPs located on CHR 16 with the highest associated signal values were annotated to the *ADAMTS12* gene. Throughout the whole-genotype patterns, distinct differences existed in two regions of *ADAMTS12* between four-horn breeds and other breeds (e.g., CHR16: 40,350,835–40,352,878 and 40,361,056–40,366,451; Figure 1G). The expression levels of *ADAMTS12* and *ADAMTS16* were reported to be related to reproductive capabilities in males, especially testicular development [26,27]. Interestingly, plasma testosterone concentration was negatively correlated with horn growth, indicating that peripheral plasma testosterone levels may play a role in regulating horn development [28]. *ADAMTS12* also plays an important role in chondrogenesis regulation [29]. The role of *ADAMTS12* in chondrogenesis was further found to be related to the chondrogenic regulatory factor parathyroid hormone-associated peptide (PTHrP) [30]. Other evidence shows that *ADAMTS12* and PTHrP can inhibit chondrocyte differentiation by inhibiting chondrocyte differentiation marker genes, such as *Col II* and *Sox9* [29]. The proliferation and differentiation of chondrocytes are extremely important to antlers, similar to horns [31,32].

Coincidentally, our analysis showed that *ADAMTS12* was enriched in several GO terms (Table S4), such as the negative regulation of chondrocyte differentiation, extracellular matrix, extracellular matrix organization, cellular response to bone morphogenetic protein (BMP) stimulus, ossification, and the ossification involved in bone maturation. The extracellular matrix provides a living environment for chondrocytes to facilitate their adhesion, proliferation, and secretion of cartilage matrix, thereby affecting cartilage growth [33]. BMP also plays an important role in the initiation of cartilage formation [34]. For example, the loss of BMP2 and BMP4 leads to serious damage to bone formation [35]. Therefore, the BMP signaling pathway is the main regulator of bone development [36]. Endochondral ossification, a process of formation for many bones, is essential for the growth and development of bones [37]. Interestingly, ossification was already reported to be an important process in the growth of horns and antlers [38,39].

**Figure 2:**
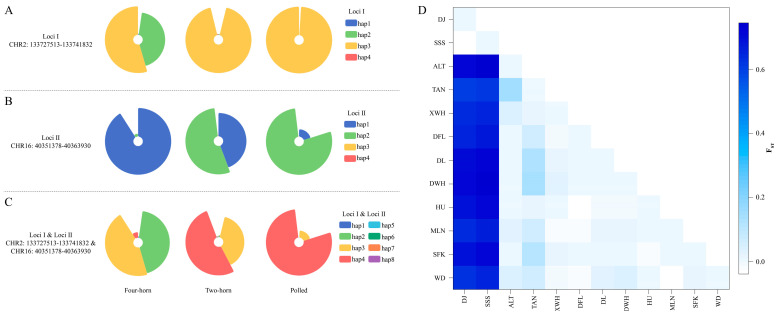
Haplotype frequency of candidate genomic region (CGR) in CHR2 and CHR16 and pairwise difference in different horn phenotype populations with CGR haplotype data. (**A**) Haplotype frequency of Loci I, (**B**) haplotype frequency of Loci II, (**C**) haplotype frequency of Loci I and Loci II, (**D**) heatmap of *F_ST_* for Loci I and Loci II among 12 breeds.

Four haplotypes were obtained from the significant SNPs in CHR2 (Loci I, Table S5) and CHR16 (Loci II, Table S6) in this study. The Hap2 and Hap3 of Loci I occupied the highest frequency (88.64%) in the FHP animals (Figure 2A), and the heterozygous individuals (Hap2/Hap3) accounted for 72.73%. Moreover, Hap1 in Loci II accounted for the highest proportion of the FHP (Figure 2B), and the homozygous individuals (Hap1/Hap1) accounted for 81.82%. Extremely significant linkage was further observed between Loci I and Loci II within 100 individuals, although they originated from different chromosomes (LD = −156.02186, *p* < 0.00001). Eight haplotypes were reconstructed from Loci I and Loci II (Table S7), with Hap2 (43.18%) and Hap3 (45.45%) having the highest frequency in the FHP (Figure 2C).

On the basis of the distributed frequency of eight haplotypes, the *F_ST_* result confirmed negligible genetic differentiation between both FHP breeds, whereas significant genetic divergence was observed between FHP breeds and others (Figure 2D and Table S9). Previous studies have hypothesized that horn inheritance is controlled by two loci, with four horns occurring if the recessive allele is homozygous at either locus [40]. The Loci I and Loci II from CHR2 and CHR16 identified in this study supported the findings of a previous one.

Moreover, although the series of SNPs did not reach a significantly associated threshold, they still had high-correlation signals distributed throughout the intergenic region between *ARNT2* and *ABHD17C* and the *HOMER2* gene in CHR18. However, studies related to *ARNT2* and *ABHD17C* have focused on the fields of cancer and immunity [41,42]. Other researchers have shown that HOMER2 is expressed in osteoblasts [43,44]. *HOMER2* and *HOMER3* can regulate bone metabolism [45]. In particular, the FoxO signaling pathway, enriched with *HOMER2*, can promote chondrocyte maturation and extracellular matrix generation [46]. Akt, also known as protein kinase B, controls the processes of endochondral ossification and skeletal growth by regulating chondrocytes and cartilage matrix by tuning the activities of FoxOs [46,47].

A previous study showed that the candidate genes of FHP in sheep are located in CHR2 and CHR10 [13]. Coincidentally, a high signal region of CHR 2 was also found in the current study. However, the high associated signal region in CHR 10 reported in a previous work was not identified here [13], but we were able to scan a novel candidate gene region (*ADAMTS12*) in CHR 16. The discrepancy could be due to the different genetic composition of the four-horn sheep group. Indeed, a private population’s genetic background can severely influence the identification of target-trait-associated markers when a single breed represents the target trait in a GWAS. Accordingly, different associated candidate genomic regions of goat wattle traits were identified from previous reports with various breeds [48,49]. Furthermore, several GWASs on sheep ear size have shown an inconsistently associated candidate genomic region because of the genetic composition of the testing animals [50,51]. Here, the richer composition of FHP sheep provides a more accurate candidate gene location.

## 4. Conclusions

GWAS was performed to identify the molecular markers and genomic regions associated with the FHP in sheep. The results confirmed that the genomic sequences from CHR2 and CHR16 contribute to this trait. This study can further elucidate the Mendelian genetic basis of the FHP in sheep.

## Data Availability

The sequence data were deposited at NCBI with the accession number PRJNA1003686.

## Supplementary Materials

The following supporting information can be downloaded at: https://www.mdpi.com/article/10.3390/ani13203166/s1, Table S1: Sample information for 100 sheep individuals. Table S2: Sequencing information of 12 Mongolian sheep. Table S3: Information on SNPs and InDels exceeding the Bonferroni significance thresholds. Table S4: Enrichment results of GO terms for genes annotated with SNPs exceeding the significance threshold. Table S5: Haplotypes for SNPs exceeding the significance threshold in CHR2. Table S6: Haplotypes for SNPs exceeding the significance threshold in CHR16. Table S7: Haplotypes for SNPs exceeding the significance threshold in CHR2 and CHR16. Table S8: Gene frequencies of SNPs in Loci I and Loci II in each breed. Table S9: Pairwise difference (*F_ST_*) in 12 different horn trait breeds with Loci I and Loci II haplotypes. Figure S1: Principal component analysis of 12 sheep breeds based on SNPs.
