# Supplementary material for: Novel Heredity Basis of the Four-Horn Phenotype in Sheep Using Genome-Wide Sequence Data
Source: Animals (Basel). 2023 Oct 10;13(20):3166. doi: 10.3390/ani13203166 (PMC10603714; doi:10.3390/ani13203166)
Supplement: Supplementary file 1 [file animals-13-03166-s001.zip › supplementary-materials/Table S2 Sequencing information of 12 Mongolian sheep.pdf]

Table S2 Sequencing information of 12 Mongolian sheep

Sample	Raw Reads	Raw Bases	Q20	Q30	GC	Sequencing Depth (fold)
DJ1	182710976	27406646400	95.36%	87.54%	42.53%	10.43
DJ2	210867810	31630171500	95.47%	87.81%	41.72%	12.04
DJ3	213119316	31967897400	95.68%	88.39%	41.94%	12.16
DJ4	201967764	30295164600	95.72%	88.45%	42.37%	11.53
DJ5	204997070	30749560500	95.91%	88.94%	42.76%	11.70
DJ6	233180350	34977052500	95.89%	88.95%	44.21%	13.31
DJ7	316067428	47410114200	95.30%	87.46%	43.00%	18.04
DJ8	241396740	36209511000	95.37%	87.55%	43.30%	13.78
DJ9	200023636	30003545400	95.46%	87.85%	42.81%	11.42
DJ10	205620270	30843040500	95.17%	87.12%	43.96%	11.74
DJ11	280191886	42028782900	95.92%	88.99%	42.92%	15.99
DJ21	229448996	34417349400	95.41%	87.71%	43.61%	13.10
