# Supplementary material for: Novel Heredity Basis of the Four-Horn Phenotype in Sheep Using Genome-Wide Sequence Data
Source: Animals (Basel). 2023 Oct 10;13(20):3166. doi: 10.3390/ani13203166 (PMC10603714; doi:10.3390/ani13203166)
Supplement: Supplementary file 1 [file animals-13-03166-s001.zip › supplementary-materials/Table S3 Information on SNPs and InDels exceeding the Bonferroni significance thresholds.pdf]

Table S3 Information on SNPs and InDels exceeding the Bonferroni significance thresholds

Variation_Type	Chromosome	Position_Info	Gene_Type	Gene_Name	*P-values*	Bonferroni corrected *P-values*
SNP	2	133,930,761	intergenic	*MTX2, HOXD1*	2.655E-17	8.967E-12
SNP	16	40,354,371	intronic	*ADAMTS12*	5.763E-16	1.928E-10
SNP	2	133,732,145	intergenic	*LOC105609047, MTX2*	7.909E-16	2.502E-10
SNP	2	133,732,461	intergenic	*LOC105609047, MTX2*	7.909E-16	2.502E-10
SNP	2	133,734,690	intergenic	*LOC105609047, MTX2*	7.909E-16	2.502E-10
SNP	2	133,737,513	intergenic	*LOC105609047, MTX2*	7.909E-16	2.502E-10
SNP	2	133,738,352	intergenic	*LOC105609047, MTX2*	7.909E-16	2.502E-10
SNP	2	133,741,832	intergenic	*LOC105609047, MTX2*	7.909E-16	2.502E-10
SNP	2	133,727,513	intergenic	*LOC105609047, MTX2*	1.077E-15	3.377E-10
SNP	16	40,363,930	intronic	*ADAMTS12*	1.168E-15	3.63E-10
SNP	16	40,351,378	intronic	*ADAMTS12*	1.504E-15	4.552E-10
SNP	16	40,352,577	intronic	*ADAMTS12*	1.504E-15	4.552E-10
SNP	16	40,354,900	intronic	*ADAMTS12*	1.504E-15	4.552E-10
SNP	2	133,736,440	intergenic	*LOC105609047, MTX2*	4.588E-15	1.377E-09
InDel	2	133,742,708	intergenic	*LOC105609047, MTX2*	2.395E-14	5.61E-09
InDel	2	133,743,939	intergenic	*LOC105609047, MTX2*	3.619E-14	7.86E-09
InDel	2	133,743,214	intergenic	*LOC105609047, MTX2*	5.493E-14	1.11E-08
