# Supplementary material for: Novel Heredity Basis of the Four-Horn Phenotype in Sheep Using Genome-Wide Sequence Data
Source: Animals (Basel). 2023 Oct 10;13(20):3166. doi: 10.3390/ani13203166 (PMC10603714; doi:10.3390/ani13203166)
Supplement: Supplementary file 1 [file animals-13-03166-s001.zip › supplementary-materials/Table S8 Gene frequencies of SNPs in Loci ó± and Loci ó≥ in each breed.pdf]

Table S8 Gene frequencies of SNPs in Loci I and Loci II in each breed

Chromosome	Position			Four-horn		Two-horn			Polled							phenotypic variance explained by SNP
				DJ	SSS	ALT	TAN	XWH	DFL	DL	DWH	HU	MLN	SFK	WD	
CHR2	133727513	Ref	C	0.625	0.500	1.000	0.833	0.900	1.000	1.000	1.000	0.950	1.000	1.000	1.000	48.270%
		Alt	T	0.375	0.500	0.000	0.167	0.100	0.000	0.000	0.000	0.050	0.000	0.000	0.000	
CHR2	133732145	Ref	T	0.625	0.500	1.000	0.833	1.000	1.000	1.000	1.000	0.950	1.000	1.000	1.000	48.592%
		Alt	G	0.375	0.500	0.000	0.167	0.000	0.000	0.000	0.000	0.050	0.000	0.000	0.000	
CHR2	133732461	Ref	G	0.625	0.500	1.000	0.833	1.000	1.000	1.000	1.000	0.950	1.000	1.000	1.000	48.592%
		Alt	A	0.375	0.500	0.000	0.167	0.000	0.000	0.000	0.000	0.050	0.000	0.000	0.000	
CHR2	133734690	Ref	C	0.625	0.500	1.000	0.833	1.000	1.000	1.000	1.000	0.950	1.000	1.000	1.000	48.592%
		Alt	T	0.375	0.500	0.000	0.167	0.000	0.000	0.000	0.000	0.050	0.000	0.000	0.000	
CHR2	133736440	Ref	C	0.636	0.500	1.000	0.833	0.900	1.000	1.000	1.000	0.950	1.000	1.000	1.000	47.080%
		Alt	T	0.364	0.500	0.000	0.167	0.100	0.000	0.000	0.000	0.050	0.000	0.000	0.000	
CHR2	133737513	Ref	T	0.625	0.500	1.000	0.833	1.000	1.000	1.000	1.000	0.950	1.000	1.000	1.000	48.592%
		Alt	A	0.375	0.500	0.000	0.167	0.000	0.000	0.000	0.000	0.050	0.000	0.000	0.000	
CHR2	133738352	Ref	A	0.625	0.500	1.000	0.833	1.000	1.000	1.000	1.000	0.950	1.000	1.000	1.000	48.592%
		Alt	G	0.375	0.500	0.000	0.167	0.000	0.000	0.000	0.000	0.050	0.000	0.000	0.000	
CHR2	133741832	Ref	G	0.625	0.500	1.000	0.833	1.000	1.000	1.000	1.000	0.950	1.000	1.000	1.000	48.592%
		Alt	T	0.375	0.500	0.000	0.167	0.000	0.000	0.000	0.000	0.050	0.000	0.000	0.000	
CHR16	40351378	Ref	G	0.083	0.100	0.300	0.667	0.550	0.600	0.750	0.833	0.750	1.000	0.857	0.667	46.724%
		Alt	A	0.917	0.900	0.700	0.333	0.450	0.400	0.250	0.167	0.250	0.000	0.143	0.333	
CHR16	40352577	Ref	G	0.083	0.100	0.300	0.667	0.550	0.600	0.750	0.833	0.750	1.000	0.857	0.667	46.724%
		Alt	A	0.917	0.900	0.700	0.333	0.450	0.400	0.250	0.167	0.250	0.000	0.143	0.333	
CHR16	40354371	Ref	C	0.083	0.100	0.300	0.667	0.550	0.600	0.750	0.833	0.750	1.000	0.857	0.750	47.641%
		Alt	T	0.917	0.900	0.700	0.333	0.450	0.400	0.250	0.167	0.250	0.000	0.143	0.250	
CHR16	40354900	Ref	G	0.083	0.100	0.300	0.667	0.550	0.600	0.750	0.833	0.750	1.000	0.857	0.667	46.724%

		Alt	A	0.917	0.900	0.700	0.333	0.450	0.400	0.250	0.167	0.250	0.000	0.143	0.333	
CHR16	40363930	Ref	T	0.083	0.100	0.300	0.667	0.500	0.600	0.750	0.833	0.750	0.929	0.857	0.667	47.036%
		Alt	G	0.917	0.900	0.700	0.333	0.500	0.400	0.250	0.167	0.250	0.071	0.143	0.333	
