# Supplementary material for: Novel Heredity Basis of the Four-Horn Phenotype in Sheep Using Genome-Wide Sequence Data
Source: Animals (Basel). 2023 Oct 10;13(20):3166. doi: 10.3390/ani13203166 (PMC10603714; doi:10.3390/ani13203166)
Supplement: Supplementary file 1 [file animals-13-03166-s001.zip › supplementary-materials/Table S9 Pairwise difference (FST) of 12 different horn trait breeds with Loci I & Loci II haplotypes.pdf]

Table S9 Pairwise difference (F_{ST}) of 12 different horn trait breeds with Loci I & Loci II haplotypes

	DJ	SSS	ALT	TAN	XWH	DFL	DL	DWH	HU	MLN	SFK	WD
DJ	0.00000											
SSS	-0.02907	0.00000										
ALT	**0.71918***	**0.74436***	0.00000									
TAN	**0.60050***	**0.61017***	0.15493	0.00000								
XWH	**0.63647***	**0.65455***	0.03509	0.01334	0.00000							
DFL	**0.65699***	**0.67818***	0.00000	0.06977	-0.01149	0.00000						
DL	**0.69757***	**0.72174***	0.00000	0.12568	0.02059	0.00000	0.00000					
DWH	**0.70880***	**0.73355***	0.00000	0.14081	0.02822	0.00000	0.00000	0.00000				
HU	**0.68740***	**0.70865***	0.00000	0.01123	0.00135	-0.03967	-0.01165	-0.00544	0.00000			
MLN	**0.64607***	**0.66791***	0.02648	0.06946	-0.01546	-0.02593	0.00986	0.01857	0.00402	0.00000		
SFK	**0.68533***	**0.70874***	0.00000	0.10923	0.01187	0.00000	0.00000	0.00000	-0.01896	0.00000	0.00000	
WD	**0.62964***	**0.65054***	0.04486	0.06061	-0.00418	-0.01617	0.02493	0.03532	0.00914	-0.03923	0.01335	0.00000

Note: * indicates significant genetic divergence between 2 breeds (P <0.05).
