# Supplementary figures and images for: Novel Heredity Basis of the Four-Horn Phenotype in Sheep Using Genome-Wide Sequence Data
Source: Animals (Basel). 2023 Oct 10;13(20):3166. doi: 10.3390/ani13203166 (PMC10603714; doi:10.3390/ani13203166)

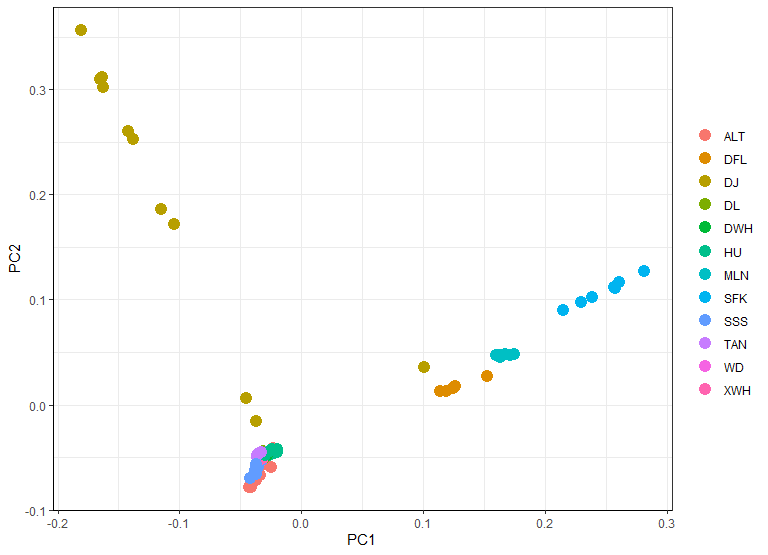

Supplement: Supplementary file 1 [file animals-13-03166-s001.zip › supplementary-materials/Figure S1 Principal component analysis for 12 sheep breeds based on SNPs.png]
